# Supplementary material for: Temporally specific engagement of distinct neuronal circuits regulating olfactory habituation in Drosophila
Source: eLife. 2018 Dec 21;7:e39569. doi: 10.7554/eLife.39569 (PMC6303106; doi:10.7554/eLife.39569)
Supplement: Supplementary file 1. [file elife-39569-supp1.docx]

**Supplementary File 1**

| **Figure 1** | | | | | | | | | | | | |
| --- | --- | --- | --- | --- | --- | --- | --- | --- | --- | --- | --- | --- |
| A. | | OCT | | ANOVA: F_(9,75)_ =29.92, p<0.0001 | | | | | | | | |
|  |  |  | | **w^1118^** | | | | | | | **w^*^** | |
|  |  | 1 min OCT | | Dunnett’s: p=0.9949 | | | | | | | Dunnett’s: p=0.9997 | |
|  |  | 2 min OCT | | Dunnett’s: p=0.7229 | | | | | | | Dunnett’s: p=0.9719 | |
|  |  | 3 min OCT | | Dunnett’s: p<0.0001 | | | | | | | Dunnett’s: p<0.0001 | |
|  |  | 4 min OCT | | Dunnett’s: p<0.0001 | | | | | | | Dunnett’s: p<0.0001 | |
|  | | | | | | | | | | | | |
| B. | |  | | ANOVA: F_(3,31)_ =36.01, p<0.0001 | | | | | | | | |
|  |  | BNZ | | **w^1118^** | | | | | | | **w^*^** | |
|  |  | 4 min BNZ | | LSM: p<0.0001 | | | | | | | LSM: p<0.0001 | |
|  | | | | | | | | | | | | |
| C. | | ETA | | ANOVA: F_(9,79)_ =22.36, p<0.0001 | | | | | | | | |
|  |  |  | | **w^1118^** | | | | | | | **w^*^** | |
|  |  | 1 min ETA | | Dunnett’s: p=0.9999 | | | | | | | Dunnett’s: p=0.9994 | |
|  |  | 2 min ETA | | Dunnett’s: p=0.7967 | | | | | | | Dunnett’s: p=0.9999 | |
|  |  | 3 min ETA | | Dunnett’s: p<0.0001 | | | | | | | Dunnett’s: p<0.0001 | |
|  |  | 4 min ETA | | Dunnett’s: p<0.0001 | | | | | | | Dunnett’s: p<0.0001 | |
|  | | | | | | | | | | | | |
| D. | | BUT | | ANOVA: F_(3,32)_ =34.6, p<0.0001 | | | | | | | | |
|  |  |  | | **w^1118^** | | | | | | | **w^*^** | |
|  |  | 4 min BUT | | LSM: p<0.0001 | | | | | | | LSM: p<0.0001 | |
|  | | | | | | | | | | | | |
| **Figure 2** | | | | | | | | | | | | |
| A. | |  | | ANOVA: F_(7,94)_ =20.18, p<0.0001 | | | | | | | | |
|  |  |  | | **w^1118^** | | | | | | | **w^*^** | |
|  |  | 1 min rest OCT | | Dunnett’s: p<0.0001 | | | | | | | Dunnett’s: p<0.0001 | |
|  |  | 3 min rest OCT | | Dunnett’s: p=0.9281 | | | | | | | Dunnett’s: p= 0.9990 | |
|  |  | 6 min rest OCT | | Dunnett’s: p=0.0551 | | | | | | | Dunnett’s: p= 0.9986 | |
|  | | | | | | | | | | | | |
| B. | |  | | ANOVA: F_(7,60)_ =22.55, p<0.0001 | | | | | | | | |
|  |  |  | | **w^1118^** | | | | | | | **w^*^** | |
|  |  | 1 min rest ETA | | Dunnett’s: p<0.0001 | | | | | | | Dunnett’s: p<0.0001 | |
|  |  | 3 min rest ETA | | Dunnett’s: p=0.0430 | | | | | | | Dunnett’s: p=0.3653 | |
|  |  | 6 min rest ETA | | Dunnett’s: p=0.7277 | | | | | | | Dunnett’s: p= 1.0000 | |
|  | | | | | | | | | | | | |
| C. | |  | | ANOVA: F_(9,75)_ =0.6856, p=0.7192 | | | | | | | | |
|  | | | | | | | | | | | | |
| D. | | 1 min OCT 0.1X-0.1X | | ANOVA: F_(2,40)_=16.10, p<0.0001  Dunnett’s: p=0.0086 | | | | | | | | |
|  |  | 4 min OCT 0.1X-0.1X | | ANOVA: F_(2,40)_=16.10, p<0.0001  Dunnett’s: p<0.0001 | | | | | | | | |
|  | | | | | | | | | | | | |
| E. | | 4 min OCT 0.1X-1X | | ANOVA: F_(2,25)_ =65.82, p<0.0001  Dunnett’s: p=0.0096 compared to naïve  LSM: p<0.0001 compared to 1X-1X | | | | | | | | |
|  |  | 4 min OCT 1X-1X | | ANOVA: F_(2,25)_ =65.82, p<0.0001  Dunnett’s: p<0.0001 | | | | | | | | |
|  | | | | | | | | | | | | |
| **Figure 3** | | | | | | | | | | | | |
| A. | |  | | ANOVA: F_(5,50)_ =72.70, p<0.0001 | | | | | | | | |
|  |  |  | | **w^1118^** | | | | | | | **w^*^** | |
|  |  | 4 min OCT | | Dunnett’s: p<0.0001 | | | | | | | Dunnett’s: p<0.0001 | |
|  |  | 4 min OCT +45V | | Dunnett’s: p=0.1412 | | | | | | | Dunnett’s: p=0.0873 | |
|  | | | | | | | | | | | | |
| B. | | 45V | | ANOVA: F_(3,35)_=1.14, p=0.3461 | | | | | | | | |
|  | | | | | | | | | | | | |
| C | |  | | ANOVA: F_(7,62)_=85.73, p<0.0001 | | | | | | | | |
|  |  |  | | **w^1118^** | | | | | | | **w^*^** | |
|  |  | 45V 0.5min before pre-exposure | | Dunnett’s: p=1.000 | | | | | | | Dunnett’s: p=1.000 | |
|  |  | 45V at the beginning of pre-exposure | | Dunnett’s: p=0.3997 | | | | | | | Dunnett’s: p=0.8711 | |
|  |  | 45V 2min after the beginning of pre-exposure | | Dunnett’s: p<0.0001 | | | | | | | Dunnett’s: p<0.0001 | |
|  |  | 45V 4.5min after the beginning of pre-exposure | | Dunnett’s: p<0.0001 | | | | | | | Dunnett’s: p<0.0001 | |
|  | | | | | | | | | | | | |
| D. | | 4 min OCT | | ANOVA: F_(2,29)_=9.83, p=0.0006  Dunnett’s: p=0.0010 | | | | | | | | |
|  |  | 4 min OCT +Vx | | ANOVA: F_(2,29)_=9.83, p=0.0006  Dunnett’s: p=0.9729 | | | | | | | | |
|  | | | | | | | | | | | | |
| E. | | Vx | | ANOVA: F_(1,35)_=0.37, p=0.5460 | | | | | | | | |
|  | | | | | | | | | | | | |
| **Figure 4** | | | | | | | | | | | | |
| A. | | shibire^ts^/+ | | ANOVA: F_(2,30)_=1.51, p=0.2375 | | | | | | | | |
|  |  | LN1Gal4>shibire^ts^ | | ANOVA: F_(2,28)_=8.61, p=0.0014 | | | | | | | | |
|  |  |  |  | **1 min OCT** | | | | | | **1 min OCT +45V** | | |
|  |  |  |  | Dunnett’s: p=0.0012 | | | | | | Dunnett’s: p=0.6268 | | |
|  | | | | | | | | | | | | |
| B. | | shibire^ts^/+ | | ANOVA: F_(2,45)_=0.79, p=0.4588 | | | | | | | | |
|  |  | GH298Gal4>shibire^ts^ | | ANOVA: F_(2,47)_=10.39, p=0.0002 | | | | | | | | |
|  |  |  |  | **1 min OCT** | | | | | | **1 min OCT +45V** | | |
|  |  |  |  | Dunnett’s: p<0.0001 | | | | | | Dunnett’s: p=0.4332 | | |
|  | | | | | | | | | | | | |
| C. | | shibire^ts^/+ | | ANOVA: F_(2,35)_=0.69, p=0.5097 | | | | | | | | |
|  |  | krasGal4>shibire^ts^ | | ANOVA: F_(2,39)_=2.27, p=0.1171 | | | | | | | | |
|  | | | | | | | | | | | | |
| D. | | shibire^ts^/+ | | ANOVA: F_(2,23)_=1.99, p=0.1609 | | | | | | | | |
|  |  | GH146Gal4>shibire^ts^ | | ANOVA: F_(2,24)_=35.41, p<0.0001 | | | | | | | | |
|  |  |  |  | **1 min OCT** | | | | | | **1 min OCT +45V** | | |
|  |  |  |  | Dunnett’s: p<0.0001 | | | | | | Dunnett’s: p<0.0001 | | |
|  | | | | | | | | | | | | |
| E. | | shibire^ts^/+ | | ANOVA: F_(2,36)_=2.86, p=0.0709 | | | | | | | | |
|  |  | APLGal4>shibire^ts^ | | ANOVA: F_(2,39)_=1.95, p=0.1573 | | | | | | | | |
|  | | | | | | | | | | | | |
| F. | | shibire^ts^/+ | | ANOVA: F_(2,33)_=0.77, p=0.4730 | | | | | | | | |
|  |  | MZ699Gal4>shibire^ts^ | | ANOVA: F_(2,33)_=0.94, p=0.4033 | | | | | | | | |
|  | | | | | | | | | | | | |
| **Figure 5** | | | | | | | | | | | | |
| A. | | w^1118^ | | ANOVA: F_(2,23)_=1.64, p=0.2175 | | | | | | | | |
|  |  | w^1118^ +HU | | ANOVA: F_(2,24)_=46.04, p<0.0001 | | | | | | | | |
|  |  |  |  | **1 min OCT** | | | | | | **1 min OCT +45V** | | |
|  |  |  |  | Dunnett’s: p<0.0001 | | | | | | Dunnett’s: p<0.0001 | | |
|  | | | | | | | | | | | | |
| B. | | Berlin | | ANOVA: F_(2,21)_=0.41, p=0.6721 | | | | | | | | |
|  |  | mbm^1^ | | ANOVA: F_(2,20)_=25.89, p<0.0001 | | | | | | | | |
|  |  |  |  | **1 min OCT** | | | | | | **1 min OCT +45V** | | |
|  |  |  |  | Dunnett’s: p<0.0001 | | | | | | Dunnett’s: p<0.0001 | | |
|  | | | | | | | | | | | | |
| C. | | shibire^ts^/+ | | ANOVA: F_(2,23)_= 0.81, p=0.4592 | | | | | | | | |
|  |  | MB247Gal4>shibire^ts^ | | ANOVA: F_(2,26)_=17.19, p<0.0001 | | | | | | | | |
|  |  |  |  | **1 min OCT** | | | | | | **1 min OCT +45V** | | |
|  |  |  |  | Dunnett’s: p<0.0001 | | | | | | Dunnett’s: p<0.0001 | | |
|  | | | | | | | | | | | | |
| D. | | shibire^ts^/+ | | ANOVA: F_(2,22)_=0.47, p=0.6326 | | | | | | | | |
|  |  | OK72Gal4>shibire^ts^ | | ANOVA: F_(2,21)_=32.87, p<0.0001 | | | | | | | | |
|  |  |  |  | **1 min OCT** | | | | | | **1 min OCT +45V** | | |
|  |  |  |  | Dunnett’s: p<0.0001 | | | | | | Dunnett’s: p<0.0001 | | |
|  | | | | | | | | | | | | |
| E. | | shibire^ts^/+ | | ANOVA: F_(2,34)_=0.71, p=0.5016 | | | | | | | | |
|  |  | OK72Gal4,MBGal80>shibire^ts^ | | ANOVA: F_(2,40)_=2.77, p=0.0752 | | | | | | | | |
|  | | | | | | | | | | | | |
| F. | | shibire^ts^/+ | | ANOVA: F_(2,19)_=0.86, p=0.4420 | | | | | | | | |
|  |  | c772Gal4>shibire^ts^ | | ANOVA: F_(2,20)_=14.23, p=0.0002 | | | | | | | | |
|  |  |  |  | **1 min OCT** | | | | | | **1 min OCT +45V** | | |
|  |  |  |  | Dunnett’s: p=0.0002 | | | | | | Dunnett’s: p=0.0005 | | |
|  |  | c739Gal4>shibire^ts^ | | ANOVA: F_(2,22)_=46.90, p<0.0001 | | | | | | | | |
|  |  |  |  | **1 min OCT** | | | | | | **1 min OCT +45V** | | |
|  |  |  |  | Dunnett’s: p<0.0001 | | | | | | Dunnett’s: p<0.0001 | | |
|  | | | | | | | | | | | | |
| G. | | shibire^ts^/+ | | ANOVA: F_(2,26)_=2.32, p=0.1194 | | | | | | | | |
|  |  | MB463BGal4>shibire^ts^ | | ANOVA: F_(2,28)_=10.21, p=0.0005 | | | | | | | | |
|  |  |  |  | **1 min OCT** | | | | | | **1 min OCT +45V** | | |
|  |  |  |  | Dunnett’s: p=0.0011 | | | | | | Dunnett’s: p=0.9842 | | |
|  | | | | | | | | | | | | |
| H. | | shibire^ts^/+ | | ANOVA: F_(2,27)_=0.07, p=0.9320 | | | | | | | | |
|  |  | MB131BGal4>shibire^ts^ | | ANOVA: F_(2,31)_=1.25, p=0.3026 | | | | | | | | |
|  | | | | | | | | | | | | |
| I. | | shibire^ts^/+ | | ANOVA: F_(2,32)_=0.81, p=0.4557 | | | | | | | | |
|  |  | VT44966>shibire^ts^ | | ANOVA: F_(2,31)_=3.01, p=0.0651 | | | | | | | | |
|  | | | | | | | | | | | | |
| **Figure 6** | | | | | | | | | | | | |
| A. | | shibire^ts^/+ | | ANOVA: F_(2,34)_=10.78, p=0.0003 | | | | | | | | |
|  |  |  |  | **4 min OCT** | | | | **4 min OCT +45V** | | | | |
|  |  |  |  | Dunnett’s: p=0.0002 | | | | Dunnett’s: p=0.4178 | | | | |
|  |  | LN1Gal4>shibire^ts^ | | ANOVA: F_(2,34)_=10.95, p=0.0002 | | | | | | | | |
|  |  |  |  | **4 min OCT** | | | | **4 min OCT +45V** | | | | |
|  |  |  |  | Dunnett’s: p<0.0001 | | | | Dunnett’s: p=0.0867 | | | | |
|  | | | | | | | | | | | | |
| B. | | shibire^ts^/+ | | ANOVA: F_(2,39)_=17.90, p<0.0001 | | | | | | | | |
|  |  |  |  | **4 min OCT** | | | | **4 min OCT +45V** | | | | |
|  |  |  |  | Dunnett’s: p<0.0001 | | | | Dunnett’s: p=0.1676 | | | | |
|  |  | krasGal4>shibire^ts^ | | ANOVA: F_(2,39)_=10.54, p=0.0002 | | | | | | | | |
|  |  |  |  | **4 min OCT** | | | | **4 min OCT +45V** | | | | |
|  |  |  |  | Dunnett’s: p<0.0001 | | | | Dunnett’s: p=0.0977 | | | | |
|  | | | | | | | | | | | | |
| C. | | shibire^ts^/+ | | ANOVA: F_(2,42)_=8.29, p=0.0010 | | | | | | | | |
|  |  |  |  | **4 min OCT** | | | | **4 min OCT +45V** | | | | |
|  |  |  |  | Dunnett’s: p=0.0026 | | | | Dunnett’s: p=0.9159 | | | | |
|  |  | GH146Gal4>shibire^ts^ | | ANOVA: F_(2,40)_=1.04, p=0.3640 | | | | | | | | |
|  | | | | | | | | | | | | |
| D. | | shibire^ts^/+ | | ANOVA: F_(2,33)_=10.28, p=0.0004 | | | | | | | | |
|  |  |  |  | **4 min OCT** | | | | **4 min OCT +45V** | | | | |
|  |  |  |  | Dunnett’s: p=0.0002 | | | | Dunnett’s: p=0.1491 | | | | |
|  |  | MZ699Gal4>shibire^ts^ | | ANOVA: F_(2,39)_=0.50, p=0.6121 | | | | | | | | |
|  | | | | | | | | | | | | |
| G. | | shibire^ts^/+ | | ANOVA: F_(2,36)_=10.64, p=0.0003 | | | | | | | | |
|  |  |  |  | **4 min OCT** | | | | **4 min OCT +45V** | | | | |
|  |  |  |  | Dunnett’s: p=0.0003 | | | | Dunnett’s: p=0.8229 | | | | |
|  |  | MB247Gal4>shibire^ts^ | | ANOVA: F_(2,41)_=6.84, p=0.0028 | | | | | | | | |
|  |  |  |  | **4 min OCT** | | | | **4 min OCT +45V** | | | | |
|  |  |  |  | Dunnett’s: p=0.0033 | | | | Dunnett’s: p=0.0075 | | | | |
|  | | | | | | | | | | | | |
| E. | uninduced | | ANOVA: F_(2,34)_=0.01, p=0.9965 | | | | | | | | | |
|  | 1 min 32C | | ANOVA: F_(2,29)_=4.00, p=0.0300 | | | | | | | | | |
|  | 4 min 32C | | ANOVA: F_(2,29)_=1.37, p=0.2707 | | | | | | | | | |
|  | | | | | | | | | | | | |
| F. | uninduced | | ANOVA: F_(2,50)_=3.49, p=0.0386 | | | | | | | | | |
|  | 1 min 32C | | ANOVA: F_(2,44)_=4.36, p=0.0190 | | | | | | | | | |
|  | 4 min 32C | | ANOVA: F_(2,49)_=13.78, p<0.0001 | | | | | | | | | |
|  |  |  | **MZ699Gal4/+** | | **MZ699Gal4>TRPA1** | | | | | | | **TRPA1/+** |
|  |  |  | Dunnett’s: p=1.0000 | | Dunnett’s: p<0.0001 | | | | | | | Dunnett’s: p=0.1898 |
|  | | | | | | | | | | | | |
| **Figure 7** | | | | | | | | | | | | |
| A. | | w^1118^ | | ANOVA: F_(2,45)_=0.64, p=0.5346 | | | | | | | | |
|  |  | rut^2080^ | | ANOVA: F_(2,47)_=8.03, p=0.0010 | | | | | | | | |
|  |  |  |  | **1 min OCT** | | | | | **1 min OCT +45V** | | | |
|  |  |  |  | Dunnett’s: p=0.0011 | | | | | Dunnett’s: p=0.7873 | | | |
|  | | | | | | | | | | | | |
| B. | | LN1Gal4/+ | | ANOVA: F_(2,31)_=1.11, p=0.3432 | | | | | | | | |
|  |  | LN1Gal4>rut^2080^;UASrut | | ANOVA: F_(2,39)_=0.11, p=0.8976 | | | | | | | | |
|  |  | rut^2080^;UASrut/+ | | ANOVA: F_(2,36)_=6.15, p=0.0053 | | | | | | | | |
|  |  |  |  | **1 min OCT** | | | | | **1 min OCT +45V** | | | |
|  |  |  |  | Dunnett’s: p=0.0079 | | | | | Dunnett’s: p=0.9907 | | | |
|  | | | | | | | | | | | | |
| C. | | LN1Gal80^ts^/+ | | ANOVA: F_(2,58)_=2.95, p=0.0606 | | | | | | | | |
|  |  | LN1Gal80^ts^>rut RNAi | | ANOVA: F_(2,57)_=10.65, p<0.0001 | | | | | | | | |
|  |  |  |  | **1 min OCT** | | | | | **1 min OCT +45V** | | | |
|  |  |  |  | Dunnett’s: p<0.0001 | | | | | Dunnett’s: p=0.4642 | | | |
|  |  | rut RNAi/+ | | ANOVA: F_(2,54)_=0.52, p=0.5997 | | | | | | | | |
|  | | | | | | | | | | | | |
| D. | | GH146Gal4/+ | | ANOVA: F_(2,33)_=0.96, p=0.3945 | | | | | | | | |
|  |  | GH146Gal4>rut^2080^;UASrut | | ANOVA: F_(2,33)_=8.43, p=0.0012 | | | | | | | | |
|  |  |  |  | **1 min OCT** | | | | | **1 min OCT +45V** | | | |
|  |  |  |  | Dunnett’s: p=0.0024 | | | | | Dunnett’s: p=0.9756 | | | |
|  |  | rut^2080^;UASrut/+ | | ANOVA: F_(2,33)_=7.58, p=0.0021 | | | | | | | | |
|  |  |  |  | **1 min OCT** | | | | | **1 min OCT +45V** | | | |
|  |  |  |  | Dunnett’s: p=0.0041 | | | | | Dunnett’s: p=1.0000 | | | |
|  | | | | | | | | | | | | |
| E. | | MB247Gal4/+ | | ANOVA: F_(2,33)_=0.60, p=0.5566 | | | | | | | | |
|  |  | MB247Gal4>rut^2080^;UASrut | | ANOVA: F_(2,31)_=13.67, p<0.0001 | | | | | | | | |
|  |  |  |  | **1 min OCT** | | | | | **1 min OCT +45V** | | | |
|  |  |  |  | Dunnett’s: p<0.0001 | | | | | Dunnett’s: p=0.8541 | | | |
|  |  | rut^2080^;UASrut/+ | | ANOVA: F_(2,32)_=6.11, p=0.0059 | | | | | | | | |
|  |  |  |  | **1 min OCT** | | | | | **1 min OCT +45V** | | | |
|  |  |  |  | Dunnett’s: p=0.0060 | | | | | Dunnett’s: p=0.8078 | | | |
|  | | | | | | | | | | | | |
| F. | | w^1118^ | | ANOVA: F_(2,47)_=9.55, p=0.0004 | | | | | | | | |
|  |  |  |  | **4 min OCT** | | | | | **4 min OCT +45V** | | | |
|  |  |  |  | Dunnett’s: p=0.0002 | | | | | Dunnett’s: p=0.1846 | | | |
|  |  | rut^2080^ | | ANOVA: F_(2,46)_=8.81, p=0.0006 | | | | | | | | |
|  |  |  |  | **4 min OCT** | | | | | **4 min OCT +45V** | | | |
|  |  |  |  | Dunnett’s: p=0.0039 | | | | | Dunnett’s: p=0.8037 | | | |
|  | | | | | | | | | | | | |
| G. | | w^1118^ | | ANOVA: F_(2,42)_=5.73, p=0.0065 | | | | | | | | |
|  |  |  |  | **30 min OCT** | | | | | **30 min OCT +YP** | | | |
|  |  |  |  | Dunnett’s: p=0.0067 | | | | | Dunnett’s: p=0.9279 | | | |
|  |  | rut^2080^ | | ANOVA: F_(2,35)_=0.87, p=0.4287 | | | | | | | | |
|  | | | | | | | | | | | | |
|  | | | | | | | | | | | | |
|  | | | | | | | | | | | | |
|  | | | | | | | | | | | | |
| **Figure 1, Supplemental Figure 1** | | | | | | | | | | | | |
| A. | | w^1118^ | | ANOVA: F_(2,42)_=0.34, p=0.7125 | | | | | | | | |
|  | | | | | | | | | | | | |
| B. | | w^1118^ | | ANOVA: F_(2,41)_=12.54, p<0.0001 | | | | | | | | |
|  |  |  |  | **4 min OCT** | | **1 min OCT x 4** | | | | | | |
|  |  |  |  | Dunnett’s: p=0.0003 | | Dunnett’s: p=0.0001 | | | | | | |
|  | | | | | | | | | | | | |
| **Figure 2, Supplemental Figure 1** | | | | | | | | | | | | |
|  | | w^1118^ | | ANOVA: F_(1,15)_=2.54, p=0.1332 | | | | | | | | |
|  |  | w^*^ | | ANOVA: F_(1,15)_=1.38, p=0.2597 | | | | | | | | |
|  | | | | | | | | | | | | |
| **Figure 4, Supplemental Figure 1** | | | | | | | | | | | | |
|  | | TTX/+ | | ANOVA: F_(2,21)_=1.34, p=0.2864 | | | | | | | | |
|  |  | GH146Gal4>TTX | | ANOVA: F_(2,24)_=52.54, p<0.0001 | | | | | | | | |
|  |  |  |  | **1 min OCT** | | **1 min OCT +45V** | | | | | | |
|  |  |  |  | Dunnett’s: p<0.0001 | | Dunnett’s: p<0.0001 | | | | | | |
|  | | | | | | | | | | | | |
| **Figure 4 Supplemental Figure 2** | | | | | | | | | | | | |
|  | | GH146Gal4/+ | | ANOVA: F_(2,31)_=3.45, p=0.0452 | | | | | | | | |
|  |  | GH146Gal4>Gad RNAi | | ANOVA: F_(2,31)_=1.80, p=0.1832 | | | | | | | | |
|  |  | Gad RNAi/+ | | ANOVA: F_(2,32)_=0.06, p=0.9395 | | | | | | | | |
|  | | | | | | | | | | | | |
| **Figure 5, Supplemental Figure 1** | | | | | | | | | | | | |
|  | | w^1118^ | | ANOVA: F_(1,16)_=0.42, p=0.5251 | | | | | | | | |
|  | | | | | | | | | | | | |
| **Figure 5, Supplemental Figure 2** | | | | | | | | | | | | |
|  | | TTX/+ | | ANOVA: F_(2,28)_=1.27, p=0.2986 | | | | | | | | |
|  |  | MB247Gal4>TTX | | ANOVA: F_(2,24)_=18.36, p<0.0001 | | | | | | | | |
|  |  |  |  | **1 min OCT** | | **1 min OCT +45V** | | | | | | |
|  |  |  |  | Dunnett’s: p<0.0001 | | Dunnett’s: p=0.0001 | | | | | | |
|  | | | | | | | | | | | | |
| **Figure 6, Supplemental Figure 1** | | | | | | | | | | | | |
|  | | GH146Gal4/+ | | ANOVA: F_(2,39)_=7.65, p=0.0017 | | | | | | | | |
|  |  |  |  | **4 min OCT** | | | **4 min OCT +45V** | | | | | |
|  |  |  |  | Dunnett’s: p=0.0008 | | | Dunnett’s: p=0.2525 | | | | | |
|  |  | GH146Gal4>Gad RNAi | | ANOVA: F_(2,39)_=10.39, p=0.0003 | | | | | | | | |
|  |  |  |  | **4 min OCT** | | | **4 min OCT +45V** | | | | | |
|  |  |  |  | Dunnett’s: p<0.0001 | | | Dunnett’s: p=0.2233 | | | | | |
|  |  | Gad RNAi/+ | | ANOVA: F_(2,38)_=9.00, p=0.0007 | | | | | | | | |
|  |  |  |  | **4 min OCT** | | | **4 min OCT +45V** | | | | | |
|  |  |  |  | Dunnett’s: p=0.0078 | | | Dunnett’s: p=0.4234 | | | | | |
|  | | | | | | | | | | | | |
| **Figure7. Supplemental Figure 1** | | | | | | | | | | | | |
| A. | | w^1118^ | | ANOVA: F_(2,46)_=9.57, p=0.0004 | | | | | | | | |
|  |  |  |  | **30 min OCT** | | **3 x 10 min OCT** | | | | | | |
|  |  |  |  | Dunnett’s: p=0.0010 | | Dunnett’s: p=0.3282 | | | | | | |
|  | | | | | | | | | | | | |
| B. | | w^1118^ | | ANOVA: F_(2,29)_=6.70, p=0.0043 | | | | | | | | |
|  |  |  |  | **30 min OCT** | | **30 min OCT**  **+ 6 min rest** | | | | | | |
|  |  |  |  | Dunnett’s: p=0.0023 | | Dunnett’s p=0.3282 | | | | | | |
|  | | | | | | | | | | | | |
| C | | w^1118^ | | ANOVA: F_(2,29)_=12.62, p<0.0001 | | | | | | | | |
|  |  |  |  | **30 min OCT** | | **30 min OCT**  **+ 30 min rest** | | | | | | |
|  |  |  |  | Dunnett’s: p<0.0001 | | Dunnett’s: p=0.0122 | | | | | | |
|  | | | | | | | | | | | | |
| D. | | TTX/+ | | ANOVA: F_(2,32)_=10.26, p=0.0004 | | | | | | | | |
|  |  |  |  | **30 min OCT** | | **30 min OCT +45V** | | | | | | |
|  |  |  |  | Dunnett’s: p=0.0012 | | Dunnett’s: p=0.0007 | | | | | | |
|  | | | | | | | | | | | | |
| E. | | TTX/+ | | ANOVA: F_(2,28)_=7.52, p=0.0026 | | | | | | | | |
|  |  |  |  | **30 min OCT** | | **30 min OCT +VX** | | | | | | |
|  |  |  |  | Dunnett’s: p=0.0039 | | Dunnett’s: p=0.0061 | | | | | | |
|  | | | | | | | | | | | | |
| F. | | TTX/+ | | ANOVA: F_(2,34)_=9.97, p=0.0004 | | | | | | | | |
|  |  |  |  | **30 min OCT** | | **30 min OCT +YP** | | | | | | |
|  |  |  |  | Dunnett’s: p=0.0003 | | Dunnett’s: p=0.4588 | | | | | | |
