# Supplementary material for: Temporally specific engagement of distinct neuronal circuits regulating olfactory habituation in Drosophila
Source: eLife. 2018 Dec 21;7:e39569. doi: 10.7554/eLife.39569 (PMC6303106; doi:10.7554/eLife.39569)
Supplement: Supplementary file 2. — Data are represented as mean ± SEM and all data are presented in Source data 1. subsequent Dunnett’s test: p=0.0016 for 1 min OCT and p=0.9918 for 1 min OCT +45V two subsequent Dunnett’s test: p=0.0003 for 1 min OCT and p=0.1084 for 1 min OCT +45V [file elife-39569-supp2.doc]

**Supplementary File 2. Driver/+ after 1-min OCT pre-exposure**

| **Genotype** | **Naïve** | **1 min OCT** | **1 min OCT +45V** | **ANOVA** |
| --- | --- | --- | --- | --- |
| LN1Gal4/+ | 74.56 ± 4.30 | 54.82 ± 4.57 | 64.27 ± 5.81 | F_(2,30)_=4.08, **p=0.0278** |
| GH298Gal4/+ | 71.62 ± 3.51 | 47.85 ± 7.09 | 62.66 ± 4.06 | F_(2,29)_=5.47,  **p=0.0101** |
| krasGal4/+ | 52.83 ± 4.45 | 27.54 ± 3.38 | 53.54 ± 6.01 | F_(2,34)_=9.20, **p=0.0007 ^1^** |
| GH146Gal4/+ | 82.71 ± 2.92 | 75.22 ± 3.83 | 74.88 ± 6.52 | F_(2,34)_=0.89,  **p=0.4209** |
| APLGal4/+ | 79.98 ± 2.99 | 62.87 ± 5.29 | 78.20 ± 4.36 | F_(2,31)_=4.64,  **p=0.0178** |
| MZ699Gal4/+ | 92.73 ± 1.70 | 78.49 ± 2.50 | 86.25 ± 2.75 | F_(2,35)_=9.14,  **p=0.0007 ^2^** |
| MB247Gal4/+ | 73.18 ± 4.75 | 62.22 ± 5.07 | 77.33 ± 3.55 | F_(2,44)_=3.13,  **p=0.0540** |
| OK72Gal4/+ | 81.31 ± 3.35 | 67.92 ± 5.44 | 71.27 ± 4.13 | F_(2,31)_=2.40,  **p=0.1086** |
| OK72Gal4/+;MBGal80/+ | 77.52 ± 4.00 | 65.89 ± 4.84 | 76.60 ± 4.06 | F_(2,39)_=2.27,  **p=0.1170** |
| C739Gal4/+ | 82.13 ± 4.46 | 69.30 ± 4.09 | 70.62 ± 2.90 | F_(2,29)_=3.32,  **p=0.0514** |
| C772Gal4/+ | 83.68 ± 3.62 | 84.12 ± 2.02 | 80.97 ± 3.55 | F_(2,29)_=0.29,  **p=0.7478** |
| MB463Gal4/+ | 76.11 ± 5.42 | 59.58 ± 5.68 | 69.98 ± 6.30 | F_(2,31)_=2.32,  **p=0.1167** |
| MB131BGal4/+ | 79.90 ± 4.33 | 64.00 ± 6.08 | 70.57 ± 4.79 | F_(2,29)_=2.38,  **p=0.1121** |
| VT44966/+ | 59.46 ± 4.70 | 43.91 ± 3.67 | 55.52 ± 3,90 | F_(2,39)_=3.79,  **p=0.0318** |
