# Supplementary material for: Temporally specific engagement of distinct neuronal circuits regulating olfactory habituation in Drosophila
Source: eLife. 2018 Dec 21;7:e39569. doi: 10.7554/eLife.39569 (PMC6303106; doi:10.7554/eLife.39569)
Supplement: Supplementary file 3. — Data are represented as mean ± SEM and all data are presented in Source data 2. subsequent Dunnett’s test: p<0.0001 for 4-min OCT and p=0.9061 for 4-min OCT +45V two subsequent Dunnett’s test: p<0.0001 for 4 min OCT and p=0.1594 for 4-min OCT +45V three subsequent Dunnett’s test: p<0.0001 for 4-min OCT and p=0.6941 for 4-min OCT +45V four subsequent Dunnett’s test: p=0.0007 for 4-min OCT and p=0.7259 for 4-min OCT +45V five subsequent Dunnett’s test: p<0.0001 for 4-min OCT and p=0.1046 for 4-min OCT +45V [file elife-39569-supp3.doc]

**Supplementary File 3. Driver/+ after 4-min OCT pre-exposure**

| **Genotype** | **Naïve** | **4 min OCT** | **4 min OCT +45V** | **ANOVA** |
| --- | --- | --- | --- | --- |
| LN1Gal4/+ | 57.73 ± 5.94 | 14.88 ± 4.84 | 55.06 ± 4.49 | F_(2,35)_=21.91,  **p<0.0001 ^1^** |
| krasGal4/+ | 54.55 ± 4.32 | 15.73 ± 4.98 | 43.64 ± 2.55 | F_(2,36)_=23.22,  **p<0.0001 ^2^** |
| GH146Gal4/+ | 77.14 ± 4.01 | 48.13 ± 5.31 | 72.62 ± 3.63 | F_(2,44)_=12.74,  **p<0.0001 ^3^** |
| MZ699Gal4/+ | 68.60 ± 4.69 | 37.74 ± 6.76 | 63.01 ± 4.54 | F_(2,36)_=8.95,  **p=0.0008 ^4^** |
| MB247Gal4/+ | 77.48 ± 4.69 | 39.75 ± 3.72 | 65.68 ± 3.99 | F_(2,45)_=23.31,  **p<0.0001 ^5^** |
