# Supplementary material for: Temporally specific engagement of distinct neuronal circuits regulating olfactory habituation in Drosophila
Source: eLife. 2018 Dec 21;7:e39569. doi: 10.7554/eLife.39569 (PMC6303106; doi:10.7554/eLife.39569)
Supplement: Transparent reporting form [file elife-39569-transrepform.docx]

***eLife’s* transparent reporting form**

We encourage authors to provide detailed information *within their submission* to facilitate the interpretation and replication of experiments. Authors can upload supporting documentation to indicate the use of appropriate reporting guidelines for health-related research (see [EQUATOR Network](http://www.equator-network.org/%20)), life science research (see the [BioSharing Information Resource](https://biosharing.org/)), or the [ARRIVE guidelines](http://www.plosbiology.org/article/info:doi/10.1371/journal.pbio.1000412) for reporting work involving animal research. Where applicable, authors should refer to any relevant reporting standards documents in this form.

If you have any questions, please consult our Journal Policies and/or contact us: [editorial@elifesciences.org](mailto:editorial@elifesciences.org).

**Sample-size estimation**

- You should state whether an appropriate sample size was computed when the study was being designed
- You should state the statistical method of sample size computation and any required assumptions
- If no explicit power analysis was used, you should describe how you decided what sample (replicate) size (number) to use

Please outline where this information can be found within the submission (e.g., sections or figure legends), or explain why this information doesn’t apply to your submission:

No explicit power analysis was used, but based on our experience in these types of analyses we aimed for enough technical replicates per genotype and condition each involving when possible, the maximum number of individual animals in an assay of at least 50. This is partially reflected in the total number of flies scored per genotype. When the total number of animals is less, escapers in the transfers within the maze or to store them account for the difference.

**Replicates**

- You should report how often each experiment was performed
- You should include a definition of biological versus technical replication
- The data obtained should be provided and sufficient information should be provided to indicate the number of independent biological and/or technical replicates
- If you encountered any outliers, you should describe how these were handled
- Criteria for exclusion/inclusion of data should be clearly stated
- High-throughput sequence data should be uploaded before submission, with a private link for reviewers provided (these are available from both GEO and ArrayExpress)

Please outline where this information can be found within the submission (e.g., sections or figure legends), or explain why this information doesn’t apply to your submission:

In this line of experiments, we aim for at least 6-12 replicates (as described above), within each experimental set. These replicates are technical.

Each experimental set was independently repeated at least twice (biological replicates) since the source of flies was an independent brood. If the first biological replicate had an n>10, the second and subsequent ones (if any) had less, typically 6-8.

**Statistical reporting**

- Statistical analysis methods should be described and justified
- Raw data should be presented in figures whenever informative to do so (typically when N per group is less than 10)
- For each experiment, you should identify the statistical tests used, exact values of N, definitions of center, methods of multiple test correction, and dispersion and precision measures (e.g., mean, median, SD, SEM, confidence intervals; and, for the major substantive results, a measure of effect size (e.g., Pearson's r, Cohen's d)
- Report exact p-values wherever possible alongside the summary statistics and 95% confidence intervals. These should be reported for all key questions and not only when the p-value is less than 0.05.

Please outline where this information can be found within the submission (e.g., sections or figure legends), or explain why this information doesn’t apply to your submission:

All ns can be inferred form the F values in the data sources, but also reported in the figure legends per figure. The statistical tests used throughout are Initial ANOVA followed by planned comparisons (Dunnett’s), or Least Square Means tests as listed in Supplemental Table 1. All ANOVA F ratios and p values as well as the p values from planned comparisons are reported in Supplemental Table 1 and all data source files. Exact p values are reported except when p<0.0001 when it listed as such.

(For large datasets, or papers with a very large number of statistical tests, you may upload a single table file with tests, Ns, etc., with reference to sections in the manuscript.)

**Group allocation**

- Indicate how samples were allocated into experimental groups (in the case of clinical studies, please specify allocation to treatment method); if randomization was used, please also state if restricted randomization was applied
- Indicate if masking was used during group allocation, data collection and/or data analysis

Please outline where this information can be found within the submission (e.g., sections or figure legends), or explain why this information doesn’t apply to your submission:

Masking was not used. We aimed for equal number of controls and experimentals generally using a balanced design with all relevant genotypes and treatments per experiment tested on the same day (n= at least 1 per genotype and/or treatment per day). When the number is not identical technical problems (flies lost, or equipment failure) accounts for the difference.

**Additional data files (“source data”)**

- We encourage you to upload relevant additional data files, such as numerical data that are represented as a graph in a figure, or as a summary table
- Where provided, these should be in the most useful format, and they can be uploaded as “Source data” files linked to a main figure or table
- Include model definition files including the full list of parameters used
- Include code used for data analysis (e.g., R, MatLab)
- Avoid stating that data files are “available upon request”

Please indicate the figures or tables for which source data files have been provided:

Source data are provided for all figures and tables as appropriately labeled excel files.
